# Unraveling the impact of customer mistreatment on highway toll collectors’ turnover intentions: the roles of stress symptoms, affective commitment, and neuroticism

**DOI:** 10.3389/fpsyg.2024.1333958

**Published:** 2024-02-19

**Authors:** Yongduan Gao, Yanyan Liu, Jianhua He, Jiaxin Zhou

**Affiliations:** ^1^Business School, Nankai University, Tianjin, China; ^2^Institute of Advanced Studies in Humanities and Social Sciences, Beijing Normal University at Zhuhai, Zhuhai, China; ^3^Management School, Beijing Normal University at Zhuhai, Zhuhai, Guangdong, China

**Keywords:** customer mistreatment, stress symptoms, affective commitment, turnover intentions, neuroticism, highway toll collectors

## Abstract

In the service industry, highway toll collectors serve as a distinctive frontline workforce who frequently encounter mistreatment from customers. Unfortunately, these behaviors have not received the attention and resolution they deserve, resulting in significant physical and psychological stress for toll collectors and exacerbating turnover rates. The study highlights how customer mistreatment affects toll collectors’ turnover intentions by performing the sequential mediating roles of stress symptoms and affective commitment and assumes that neuroticism exacerbates the stress symptoms resulting from customer mistreatment based on affective events theory. The model was tested using data collected from 230 highway toll collectors in Zhuhai, China. All hypotheses received support. This study holds both theoretical and practical implications for future research.

## 1 Introduction

Customer mistreatment, including behaviors such as condescending language and yelling directed at service employees (Dormann and Zapf, 2004; Skarlicki et al., 2008), is a kind of low-quality interpersonal treatment that employees receive from customers (Bies, 2001). As a pervasive issue, customer mistreatment exists in various service organizations (Pu et al., 2022), such as hospitality and tourism (Jung and Yoon, 2018; Wang et al., 2023), healthcare (Wu et al., 2019; Mostafa, 2022; Zhang et al., 2023) and call centers (Van Jaarsveld et al., 2021; O'Brady et al., 2023). Evidence consistently shows that this behavior is associated with a wide range of negative consequences for both employees and organizations (Men et al., 2022; Wu et al., 2023). Besides negatively affecting service employee’s affective, attitudinal and behavioral outcomes, such as employee well-being (Baranik et al., 2017; Wang and Wang, 2017; Yang et al., 2023), job satisfaction (Bamfo et al., 2018), organizational commitment (Harris, 2013; Yang and Lau, 2019), and turnover intention (Han et al., 2016), it also leads to employees’ poor performance in organizations, such as reduced service performance (Baranik et al., 2017; Park and Kim, 2020), reduced customer-directed organizational citizenship behaviors (Amarnani et al., 2022), and increased workplace deviance (Chen and Wu, 2022).

Although customer mistreatment has various negative effects towards employees, this study primarily focuses on its impact on employee turnover intentions. On one hand, this is due to the relatively limited research conducted thus far on the relationship between customer mistreatment and employee turnover intention (Han et al., 2016; Alola et al., 2019; Pu et al., 2022), and further research is required to delve into the mechanisms between them. For example, Alola et al. (2019) demonstrated emotional exhaustion mediates the effect of customer incivility and hotel employees’ turnover intentions building on conservation of resources theory. Han et al. (2016) applied the conservation of resources theory to examine the roles of employee burnout, organizational and supervisory support on the relationships between customer incivility and restaurant employees’ turnover intention. Pu et al. (2022) explored the mediating roles of emotion exhaustion, job satisfaction, and professional identity between customer mistreatment and turnover intention among hotel employees, drawing on the conservation of resources theory and the cognitive-affective personality system theory. Nevertheless, affective events theory (Weiss and Cropanzano, 1996), serving as a fundamental framework, has elucidated the influence of workplace incidents (i.e., customer mistreatment) on employees’ attitudinal and behavioral outcomes by acting as triggers for their affective responses (Koopmann et al., 2015; Wu et al., 2023), but has not been addressed in the context of explicating the relationship between customer mistreatment and employee turnover intention. On the other hand, it is a common phenomenon for service employees to experience higher frequencies of turnover intention than employees who perform standard work due to dealing with different guest issues on a daily basis (Olugbade and Karatepe, 2019; Yu et al., 2021), especially for those who are required to work in shifts (Shi et al., 2021), such as highway toll collectors, an ubiquitous yet overlooked group. Hence, this research endeavors to investigate the influence of customer mistreatment on turnover intention among highway toll collectors employing the theoretical framework of affective events.

According to affective event theory (AET) (Weiss and Cropanzano, 1996), discrete work emotional events shape employees’ work behaviors through their affective reactions and work attitudes responses (Wegge et al., 2010; Zhan et al., 2016; Baranik et al., 2017), while employees’ personality traits modify these processes (Chi et al., 2013). Past research indicates customer mistreatment can serve as the initial negative emotional event (Koopmann et al., 2015; Lee et al., 2020). Namely, when toll collectors experience rude, disrespectful, or hostile behavior from customers, it can lead to immediate negative emotional responses (Wu et al., 2023), such as anger, frustration, or sadness (Rupp and Spencer, 2006; Spencer and Rupp, 2009), resulting in psychological stress (i.e., stress symptoms) (Lovibond and Lovibond, 1995; Henry and Crawford, 2005). As Podsakoff et al. (2007) demonstrated, undesirable stress will ultimately translate into coping in the form of emotional and physical withdrawal from work, exhibiting low affective commitment and a propensity to leave. Affective commitment reflects the desire to continue employment (Vandenberghe and Bentein, 2009), which is strongly related to turnover intentions and actual turnover (Meyer et al., 2002). Therefore, in the present study, we employed the perspectives of AET to investigate the sequential mediating role of stress symptoms and affective commitment in the relationship between customer mistreatment and turnover intention. In addition, employees’ personality traits, i.e., neuroticism, have been applied to elucidate when the detrimental effect of customer negative work events experienced by hairstylists exacerbated or mitigated (Chi et al., 2013). We predict that toll collectors with higher levels of neuroticism may be more sensitive to customer mistreatment than those with lower levels of neuroticism, and thus undergo stronger stress symptoms. Therefore, the moderating role of neuroticism in those relationships was examined among toll collectors.

**Figure 1:**
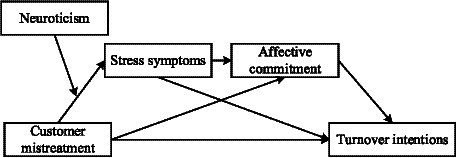
Conceptual model.

This study contributes to the existing literature through several theoretical contributions. Firstly, despite existing research examining the mediating mechanisms of customer mistreatment on turnover intention among service industry workers through the lens of conservation of resources theory (Pu et al., 2022; Salem et al., 2023), there remains a dearth of scrutiny regarding the relationship between customer mistreatment and turnover intention from a more nuanced affective perspective. This study addresses this gap by interpreting the impact of customer mistreatment on turnover intention through the lens of AET. Our study conceptualizes customer mistreatment as an event that toll collectors often experience at work, not only enriching the perspective of interpreting the relationship between customer mistreatment and turnover intention but examining the roles of stress symptoms and affective commitment in this process. Secondly, this study enriches the boundary conditions of the relationship between customer mistreatment and turnover intentions by introducing neuroticism, thereby enhancing the understanding of individual characteristics in the context of the impact of customer mistreatment. Lastly, by focusing on highway toll collectors, this study empirically extends the scope of impact of customer mistreatment within the service industry, providing insights into the effects on a specific occupational group. Besides, most research has focused on the impact of the working environment on highway toll collectors’ physical health (Cheng et al., 2010; Lai et al., 2021), thus this study marks the inaugural attention to psychological health of highway toll collectors. The conceptual model of this study is shown in Figure 1.

## 2 Literature review and hypothesis development

### 2.1 Affective events theory

AET posits that affective reactions in the workplace are shaped by external events, which, in turn, influence employees’ attitudinal and behavioral outcomes. This theoretical framework, as proposed by Weiss and Cropanzano (1996), suggests that work events serve as proximal causes for employees’ affective reactions. Positive events, such as receiving praise from a supervisor, can elicit positive affectivity (Cropanzano et al., 2017), while negative events, like customer mistreatment, can lead to negative affective responses (Wu et al., 2023). These affective reactions, in turn, play a crucial role in shaping employees’ overall attitudes towards their work and influencing their subsequent behaviors. Previous studies have confirmed that positive events tend to enhance employees’ job satisfaction, job performance, and job evaluations (Groth et al., 2019; Salem et al., 2023), while negative events can result in reduced emotional commitment (Harris, 2013; Tillman et al., 2018), adverse physical health symptoms (Wang and Wang, 2017), negative emotions (Zhan et al., 2016), reduced service performance (Babalola et al., 2019) and withdrawal behaviors at work (Ashkanasy et al., 2014; Tillman et al., 2018). Therefore, in the context of service interactions, employees may perceive negative events, such as customer mistreatment, as triggers for negative affectivity.

Specifically, AET has been identified as a crucial explanatory framework for understanding the repercussions of customer mistreatment on employee outcomes (Koopmann et al., 2015). In the context of customer mistreatment, which is regarded as a detrimental work event, the focus shifts from the mistreatment episode itself to the employees’ affective responses triggered by the experience. It is these negative affective reactions in service employees that ultimately contribute to adverse effects on their work attitudes and behaviors. In support of the AET, prior empirical research has consistently demonstrated that frontline service employees perceive episodes of customer mistreatment as significant sources of negative affective experiences. For instance, Yue et al. (2022) found a significant association between customer mistreatment and the manifestation of negative moods among service employees. Additionally, Poddar and Madupalli (2012) contended that customer mistreatment serves as a predictor of heightened stress levels among service employees. As also indicated by Grandey et al. (2004), Rupp and Spencer (2006), and Rupp et al. (2008), customer mistreatment is described as an affective event that triggers strong negative affective states in employees, which exert a detrimental influence on the attitudes and behaviors of service employees, underscoring the importance of addressing and mitigating the impact of customer mistreatment within the service industry.

Moreover, the impact mechanisms of affective events on emotional, attitudinal, and behavioral responses are contingent upon employees’ personality traits (Chi et al., 2013). Individuals with higher positive affect traits tend to be more sensitive to positive events, thus generating more positive affective responses, whereas those with higher negative affect traits exhibit the opposite tendency (Weiss and Cropanzano, 1996). Previous empirical evidence has elucidated the moderating effects of personality traits on the mechanisms of these work-related events. For example, neuroticism has been shown to intensify the indirect effects of customer negative events on employee service sabotage through affective reactions of the service worker (Chi et al., 2013). Chi et al. (2018) also confirmed that personal factors (e.g., core self-evaluations) assist service workers to alleviate the negative chain effects of customer mistreatment on work withdrawal behaviors and work–family conflict.

Therefore, aligning with the main mechanisms of the AET, this paper investigates how customer mistreatment affects toll collectors’ turnover intentions through its sequential influence on their affective states (i.e., stress symptoms) and work attitudes (i.e., affective commitment), and identify the moderating role of neuroticism in this process.

### 2.2 Customer mistreatment and toll collectors’ turnover intention

Customer mistreatment, as negative events, refers to the low-quality treatment that employees receive from customers (Wang et al., 2011). It is prevalent in industries where employees provide services and interact with customers, often manifesting as disrespectful language, shouting, or raising one’s voice towards employees (Grandey et al., 2007). It represents a form of low-quality interpersonal interaction and serves as a typical source of workplace stress, indicating the fact that employees are not being treated with respect and friendliness. Customer mistreatment is also referred to as customer incivility (Sliter et al., 2012), customer injustice (Rupp and Spencer, 2006), and customer aggression (Kumar Madupalli and Poddar, 2014). Despite the differences in terminology, these concepts convey the similar underlying content (Wilson and Holmvall, 2013). We propose that customer mistreatment will induce employees’ turnover intention. We incorporate the intention to leave instead of actual turnover since the intention to leave has been identified as the most reliable predictor of actual turnover (Maertz Jr and Griffeth, 2004). Turnover intentions represent employees’ conscious and deliberate desire to leave the organization (Sharma and Tiwari, 2023). High turnover intentions may cause significant decline in performance and substantial increase in cost, especially in service industry (Kraemer et al., 2017).

Hershcovis (2011) contends that workplace incivility and bullying exert a considerable influence on both job satisfaction and turnover intention. Subsequent to contending with instances of customer mistreatment, employees manifest psychological shifts and adopt a pessimistic stance towards their work. Baker and Kim’s (2020) survey reveal that 38% of participants express a proclivity to resign as a means of extrication from such circumstances. The impact of customer mistreatment on turnover intention is corroborated across diverse occupational domains, including tour guides (Karatepe et al., 2009), restaurant workers (Han et al., 2016), and hotel staff (Alola et al., 2019; Pu et al., 2022). Consequently, the encounter with customer mistreatment among toll collectors is posited as a catalyst for heightened turnover intentions.

Highway toll collectors work relatively independently and seldom communicate with others for the sake of job responsibility. What’s more, they are not even allowed to use mobiles during worktime. Their interaction with drivers is their main opportunity to communicate. However, in situations of prolonged waiting or poor mood, drivers may vent their anger towards them and yell at them. Even worse, toll collectors, confronted with such unfair treatment, often find it challenging to voice their concerns or defend on an equal footing with customers, considering the prevalent philosophy of “customer-centricity” and worrying about their performance appraisal (Shah et al., 2006; Habel et al., 2020). Consequently, unsatisfied with both the event and the organization, they may perceive leaving the current work environment as the relatively viable solution, thus fostering turnover intentions. Thus, we predict that toll collectors facing customer mistreatment may choose to resign. This viewpoint also aligns with the assumptions of the AET (Weiss and Cropanzano, 1996), which state that work events have significant influence on employees’ behaviors. Hence, the following hypothesis is proposed:

*Hypothesis 1*: Customer mistreatment is positively associated with toll collectors’ turnover intentions.

### 2.3 The mediating role of stress symptoms

Stress symptoms include feelings of tension, anxiety, and irritability, fall within the realm of negative affectivity as they involve emotional and psychological responses to stressors or challenging situations (Watson et al., 2014), and vary across one’s daily interactions with others (Park et al., 2020). Henry and Crawford (2005) also defined stress and negative affectivity as synonymous. In the workplace, service employees often encounter mistreatment from customers, such as disrespect or aggressive demands (Sommovigo et al., 2020). When employees frequently engage in unpleasant social interactions with customers, it leads to intense negative affectivity, including discouragement (Zhan et al., 2016), anger, anxiety, and irritability (Lim et al., 2008), that is stress symptoms. Previous studies have indirectly examined stress as outcomes of customer mistreatment (Koopmann et al., 2015; Cho et al., 2016; Yang et al., 2020), and even scholars conceptualized customer mistreatment as a daily work hassle that could induce stress for employees (Sliter et al., 2012; Zhan et al., 2016). Specifically, Dormann and Zapf (2004) classified customer mistreatment as a social stressor because it has the potential to undermine service employees’ self-efficacy, goal attainment, and available resources (also see Penney and Spector, 2005). Similarly, Wegge et al. (2010) conducted an experiment manipulating customer behavior towards call center employees, revealing that employees dealing with unfriendly customers, as opposed to friendly ones, reported heightened levels of psychological strain.

For toll collectors, when confronted with customer mistreatment in the workplace, although feel angry and have other intense stress, they usually choose to endure it instead of reasoning with customers or making a complaint about the matter. This is due to the simple fact that the relationship between toll collectors and customers is not fair, and the management usually tends to sacrifice their employees’ feelings for the sake of company image even if they lodge a complaint (Grandey et al., 2010; Jerger and Wirtz, 2017). Thus, considering the service rules and worrying about their performance appraisal, salary and employment opportunity, they usually choose to suppress their stressful feelings. In other words, the stress symptoms aroused by customer mistreatment are not addressed in time, which we propose may further activate toll collectors’ following intention to leave.

According to AET, work events elicit both proximal (e.g., affective) and distal (e.g., attitudinal) reactions, which can be disentangled (Weiss and Cropanzano, 1996). As highlighted by Tillman et al. (2018), work events serve as proximal triggers for affective reactions, and these affective experiences establish a direct connection to various work outcomes, attitudes, and behaviors. We propose that customer mistreatment, as a negative workplace event, which may manifest as uncivil or aggressive behavior from customers, can burden employees’ stress symptoms and further trigger employees’ intention to leave the organization. Specifically, the stress symptoms induced by customer mistreatment, such as increased tension, anxiety, and psychological strain, can lead to a heightened intention to resign as employees seek to alleviate the emotional toll and restore a more positive work environment (Saks and Ashforth, 1997; Avey et al., 2009). Much research indicates that that front-line employees experiencing high levels of stress not only exhibit higher levels of work withdrawal behaviors (Sliter et al., 2012), increased work deviance behaviors (Bennett and Robinson, 2000) and lower job satisfaction (Klassen and Chiu, 2010), but also have a higher propensity for resignation and actual turnover (Podsakoff et al., 2007; Park and Kim, 2020). Thus, this study suggests that customer mistreatment indirectly influences turnover intention by triggering stress symptoms in toll collectors. The following hypothesis is proposed:

*Hypothesis 2*: Stress symptoms mediate the relation between customer mistreatment and toll collectors’ turnover intention.

### 2.4 The mediating role of affective commitment

Typical customer mistreatment includes customers’ verbal abuse, unfair demands, and disrespectful behaviors (Skarlicki et al., 2008). Toll collectors often are exposed to various forms of customer mistreatment due to the nature of their job and the potential stress associated with toll booth interactions, such as verbal abuse (e.g., offensive language, insults, or shouting from frustrated or irate customers, especially during peak traffic times or when customers are in a hurry), refusal to pay (e.g., argue about the toll amount, leading to confrontations with toll collectors), and aggressive behavior (e.g., banging on windows, honking horns excessively, or engaging in threatening gestures). According to Weiss and Cropanzano‘s (1996) perspective, work events have both affective and attitudinal reactions, and these can be disentangled (also see Tillman et al., 2018). Consistent with AET, we propose that customer mistreatment as a negative work event that elicits employees’ negative affective attitudes, such as reducing affective commitment.

Specifically, affective commitment, referring to the emotional attachment existing between an employee and their organization (Meyer and Allen, 1991), is the one of most used affective work attitudes in organizational behavior research (Mignonac and Herrbach, 2004). In service research, there are two studies that have indirectly confirmed the relationship between customer mistreatment and affective commitment by sampling different service groups. For example, Yang and Lau (2019) discovered that customer incivility led to a decline in organizational commitment among hospitality frontline employees. Likewise, a study involving call center workers revealed that customer phone rage was associated with a decrease in employees’ organizational affective commitment (Harris, 2013).

Furthermore, AET also proposes that affective attitudes to work events will subsequently influence judgment-driven behaviors, such as turnover intentions (Glasø et al., 2010; Tillman et al., 2018). In this study, we predict that reduced affective commitment by customer mistreatment will induce the intention to leave an organization. Affective commitment has been shown to be the best predictor of intention to turnover (Mignonac and Herrbach, 2004). Numerous studies have also substantiated the proposition that affective commitment and turnover intentions are closely interconnected constructs (Meyer et al., 2002; Galletta et al., 2011; Tillman et al., 2018). Moreover, previous studies also found that employees with high affective commitment tend to have higher job satisfaction (Marique and Stinglhamber, 2011), greater loyalty (Meyer et al., 2002), and engage in more altruistic behaviors to express their appreciation for the organization’s values (Loi et al., 2012). These results also prove the relationship between customer mistreatment and employees’ turnover intention from the opposite side.

The negative relation between negative work events and affective commitment has been documented in existing literature (Glazer et al., 2004; Tillman et al., 2018), as has the correlation between negative work events (i.e., customer treatment) and turnover intention (Van Jaarsveld et al., 2021; Yang et al., 2023). Past articles increasingly emphasize the use of affective processes as mediators to explain these relationships (Chi et al., 2013; Craig et al., 2013). Using affective commitment as a mediating variable enables us to understand the process from experiencing customer mistreatment to having turnover intention. Consistent with AET, the model proposed in the current study hypothesizes that customer mistreatment, serving as a workplace event toll collectors experienced, is associated with negative affective attitude (i.e., via decreased affective commitment), and that this affective attitude mediates the effects of customer mistreatment on toll collectors’ turnover intention. Hence, the following hypothesis is proposed:

*Hypothesis 3*: Affective commitment mediates the relation between customer mistreatment and toll collectors’ turnover intention.

### 2.5 The mediating effect of stress symptoms and affective commitment

Within the theoretical framework of AET (Weiss and Cropanzano, 1996), work events trigger both proximal (i.e., affective) and distal (i.e., attitudinal) reactions, both of which can be disentangled (also see Tillman et al., 2018). Experiencing certain work events leads to affective states, which directly influence work attitudes and in turn both attitudes and affective states determine behavioral responses (Weiss and Cropanzano, 1996; Ohly and Schmitt, 2015). In other words, AET underscores the role of affective response in the formation of work attitudes (Carlson et al., 2011). Thus, we believe that stress symptoms and affective commitment sequentially mediate the relationship between customer mistreatment toll collectors’ turnover intention.

Specifically, previous research indicated that stress triggered by negative work events is a kind of affective state that can be experienced at work (Mignonac and Herrbach, 2004), manifesting such as frustration or anger. Commitment was defined conventionally as an individual’s attitude towards the organization, encompassing “a strong belief in, and acceptance of, an organization’s goals, willingness to exert considerable effort on behalf of the organization and a strong desire to maintain membership in the organization” (Mowday et al., 1979). As a dimension of organizational commitment, affective commitment is therefore as an attitude and not as affect (Mignonac and Herrbach, 2004). Moreover, from existing empirical research, in addition to Mignonac and Herrbach (2004) supporting the direct relationship between stress symptoms and affective commitment, Li et al. (2010) found the negative association between chaotic emotions (i.e., anxiety, fear, and upset) and organizational commitment by surveying eight Chinese firms. Therefore, stress symptoms serving as affective state can mediate the relationship between customer mistreatment and affective commitment. Combined with H2 and H3, this study thus proposes the following hypothesis:

*Hypothesis 4*: There is sequential mediation from customer mistreatment to toll collectors’ turnover intentions through stress symptoms and affective commitment.

### 2.6 The moderating role of neuroticism

AET proposes that experiencing work events leads to employees’ affective states, which process may be influenced by employees’ personality traits (Weiss and Cropanzano, 1996). We argue that toll collectors with high levels of neuroticism are more likely to respond strongly to customer mistreatment due to their heightened sensitivity to negative emotions and stressors (Wang and Wang, 2017). Neuroticism is a personality trait characterized by emotional instability, anxiety, and a tendency to experience negative emotions more intensely (Nasurdin et al., 2005). In other words, individuals with high neuroticism levels may be more reactive and less resilient in the face of mistreatment, leading to stronger emotional responses, such as frustration, anger, or distress. This heightened reactivity may be a result of their predisposition to perceive and process negative stimuli more intensely than individuals with lower neuroticism levels (Yeatts et al., 2017).

Prior research in service industry indicates that people who score high on neuroticism are more likely to experiencing heightened feelings of hopelessness, and facing an increased susceptibility to developing mood disorders, whereas those with lower neuroticism scores typically cope better with negative work events (Yeatts et al., 2017). For example, Chi et al. (2013) showed that hairstylists’ neuroticism moderated the relationship between negative events and state hostility. Davila et al. (2003) found that neuroticism intensifies the negative connection between relational conflict and depression. Hutchinson and Williams (2007) concluded that the link between daily hassles and depressive symptoms is significantly stronger among individuals with high levels of neuroticism. Jahanzeb et al. (2020) revealed that neuroticism amplifies the impact of workplace bullying on employee anger. Sliter and Jones (2016) stated that neurotic service employees, exhibiting symptoms of anxious and easily upset, have been found to report more customer incivility.

Besides, individuals with high levels of neuroticism tend to display biased negative cognitions in response to stressful situations, because of their thought patterns and interpretations of events tend to be pessimistic and skewed towards the negative (Lakdawalla and Hankin, 2008). In addition, individuals high in neuroticism are more likely to ruminate on negative cognitions, which in turn is associated with higher levels of depressive symptomology (Lam et al., 2003). It is mainly due to high neuroticism levels that can result in a tendency to perceive and amplify negative aspects of experiences, leading to prolonged and intense dwelling on distressing thoughts. In light of this, we propose that neuroticism exacerbates the effect of customer mistreatment on toll collectors’ stress symptoms. Moreover, in combination with the mediating effect of stress symptoms and affective commitment, we predict that neuroticism triggers the translation of customer mistreatment into toll collectors’ turnover intentions via increased stress symptoms and subsequently reduced affective commitment. Thus, this study presents the following hypotheses:

*Hypothesis 5a*: Neuroticism moderates the positive relationship between customer mistreatment and toll collectors’ stress symptoms such that the positive relationship is more pronounced among toll collectors in high levels of neuroticism.

*Hypothesis 5b*: Neuroticism moderates the indirect relationship between customer mistreatment and toll collectors’ turnover intentions through stress symptoms and affective commitment such that the indirect relationship will be stronger for toll collectors in high levels of neuroticism.

## 3 Methodology

### 3.1 Sample and procedure

We have obtained data for a consulting project based on our collaboration with a highway company. This survey was conducted through the human resources department manager of a highway operation company in Zhuhai, China. A questionnaire survey was administered to 274 toll collectors, with 230 valid questionnaires returned, resulting in a response rate of 83.94%. The highway operation company typically assigns an average of 3–5 toll collectors at each toll station, with an average of one toll station approximately every 10 kilometers. Therefore, the 230 toll collectors surveyed cover approximately 600 kilometers of the highway, making the sample highly representative for a city. Among the survey participants, 117 were male, accounting for 51%, while 113 were female, accounting for 49%. Regarding their organizational tenure, 138 participants had been employed for less than 3 months, constituting 60% of the sample (indicating a relatively high turnover rate). There were 27 individuals with 3–6 months (12%), 22 with 6–12 months (10%), and 43 with over 1 year (19%). When considering the cumulative job tenure, 101 participants had less than 1 year of experience (44%), 48 had 1–2 years (21%), 26 had 2–3 years (11%), 33 had 3–5 years (14%), and 22 had more than 5 years of experience (10%). In terms of educational background, 3 individuals had education at or below the junior high school level (1%), 147 had completed high school/technical school/vocational education (64%), 75 had obtained college degrees (33%), and 5 had bachelor’s degrees or higher (2%).

### 3.2 Measures

Adhering to Brislin’s (1980) translation-back-translation procedure, the English measures that had been previously established were rendered into Chinese with the assistance of two PhD specializing in the field of organizational behavior. Unless specified otherwise, respondents used a five-point Likert-type scale, where a rating of 1 represents “strongly disagree” and 5 signifies “strongly agree.”

#### 3.2.1 Customer mistreatment

The scale used in this study is derived Wang et al. (2011). Eight items relevant to the context of toll collectors’ work scenarios were selected from the scale by the manager of surveyed company. These items were used to measure the frequency of participants’ experiences of customer mistreatment during their work. Participants’ responses ranged from 1 to 5, with 1 indicating “never occurred” and 5 indicating “always occurred.” An example item is “yelled at me loudly.” The Cronbach’s α for this scale was 0.95.

#### 3.2.2 Neuroticism

Toll collectors rated their own neuroticism levels based on Donnellan et al. (2006)‘s four-item scale. An example item is “I frequently experience mood swings.” The Cronbach’s α for this scale was 0.89.

#### 3.2.3 Stress symptoms

Toll collectors were asked to report their stress symptoms in the past week using Henry and Crawford (2005)‘s seven-item scale, which revised based on the scale from Lovibond and Lovibond (1995). An example item from the scale is “I find myself getting easily annoyed.” The Cronbach’s α for this scale was 0.89.

#### 3.2.4 Affective commitment

Toll collectors were required to report their affective commitment using Meyer and Allen (1991)‘s five-item scale. An example item is “I am pleased to be a part of this organization.” The Cronbach’s α coefficient for this scale was 0.95.

#### 3.2.5 Turnover intentions

Toll collectors reported their own turnover intentions with a three-item scale from Kelloway et al. (1999). An example item from the scale is “I intend to seek an alternative job.” The Cronbach’s α for this scale was 0.92.

#### 3.2.6 Control variables

In this study, demographic variables including age, gender, education, organizational tenure and job tenure were selected as control variables in accordance with previous studies on service employees’ customer mistreatment (Chen and Francesco, 2000; Sliter et al., 2010). The observed result pattern remained consistent irrespective of the inclusion or exclusion of these control variables, as indicated by our analyses. In order to ensure transparency in reporting while maintaining conciseness, we present the means, standard deviations (SDs), and correlations of the control variables with the study variables in Table 1. The main findings are reported without the incorporation of these control variables.

**Table 1:** Descriptive and correlational analysis.

	*M*	SD	1	2	3	4	5	6	7	8	9	10
1. Age	22.56	4.09										
2. Gender	1.49	0.50	−0.17^**^									
3. Job tenure	4.28	2.60	0.18^**^	−0.14^*^								
4. Organizational tenure	2.93	1.71	0.69^**^	−0.15^*^	0.43^**^							
5. Educational level	3.35	0.59	0.40^**^	0.00	−0.02	0.21^**^						
6. Customer mistreatment	2.62	0.98	0.03	−0.01	0.21^**^	0.05	0.08	**0.83**				
7. Neuroticism	2.44	0.96	−0.08	0.01	−0.10	−0.22^**^	0.02	0.31^**^	**0.82**			
8. Stress symptoms	2.18	0.88	−0.01	0.05	0.03	−0.05	0.00	0.41^**^	0.64^**^	**0.75**		
9. Affective commitment	3.62	0.86	0.01	0.01	0.07	0.07	−0.12	−0.43^**^	−0.38^**^	−0.41^**^	**0.89**	
10. Turnover intentions	2.63	0.97	−0.07	0.08	−0.11	−0.15^*^	−0.03	0.19^**^	0.40^**^	0.31^**^	−0.53^**^	**0.90**

*N* = 230. Values in bold along the diagonal are Cronbach’s alphas. Age was calculated using the actual date of birth. Gender was code with 1 for men and 2 for women. Job tenure was coded with 1 for less than 1 year of experience, 2 for 1–2 years, 3 for 2–3 years, 4 for 3–5 years, and 5 for more than 5 years of job tenure. Organizational tenure was coded with 1 for less than 3 months, 2 for 3–6 months, 3 for 6–12 months, and 4 for over 1 year. Education was coded with 1 for the junior high school level and below, 2 for high school/technical school/vocational education, 3 for college degrees, and 5 for bachelor’s degrees and above. ^*^p < 0.05, ^**^p < 0.01.

## 4 Results

### 4.1 Confirmatory factor analysis

When assessing the validity of a questionnaire using factor analysis, it is first necessary to satisfy the assumptions of factor analysis, which means that the items should exhibit strong correlations. Initially, we conducted Kaiser-Meyer-Olkin (KMO) and Bartlett’s sphericity tests using SPSS 26.0 to validate the overall questionnaire. The KMO value was 0.917, and Bartlett’s Test of Sphericity yielded an approximate chi-square value of 5185.41 (*df* = 351, *p* = 0.00). These results indicate that the data meet the prerequisites for factor analysis, and the questionnaire demonstrates good validity, making it an effective instrument.

A set of confirmatory factor analyses (CFA) were performed to verify the discriminant validity of our focal variables using Amos 26.0 (see Table 2). Among all the alternative models, the five-factor model demonstrates a significantly better fit (χ^2^/df = 1.76, RMSEA = 0.06, SRMR = 0.04, CFI = 0.95, IFI = 0.95, TLI = 0.95) compared to the other four competitive models. Thus, the model variables selected in this study have good discrimination and are suitable for further analysis.

**Table 2:** Confirmatory factor analysis for discriminant validity.

Model	χ2	df	χ2/df	RMSEA	SRMR	CFI	IFI	TLI
Five-factor model (CM, NE, SS, AC, TI)	553.44^**^	314	1.76	0.06	0.04	0.95	0.95	0.95
Four-factor model (CM, NE + SS, AC, TI)	758.45^**^	318	2.39	0.08	0.05	0.91	0.91	0.90
Three-factor model (CM, NE + SS, AC + TI)	1152.84^**^	321	3.59	0.11	0.07	0.84	0.84	0.82
Two-factor model (CM + NE + SS, AC + TI)	2153.95^**^	323	6.67	0.16	0.16	0.64	0.64	0.61
One-factor model (CM + NE + SS + AC + TI)	3107.78^**^	324	9.59	0.19	0.16	0.45	0.45	0.41

Variables were merged based on their similarity to each other. CM, customer mistreatment; NE, neuroticism; SS, stress symptoms; AC, affective commitment; TI, turnover intentions, “+” indicates factor combination; ^*^p < 0.05, ^**^p < 0.01.

### 4.2 Harman’s one-factor test

In the process of collecting data through surveys, researchers can be susceptible to the potential bias introduced by the way questions are framed, as well as factors related to the emotions of the research subjects and the overall survey environment. This susceptibility can lead to Common Method Bias. To assess whether this issue is significant in our study, we employed Harman’s one-factor test analysis method. The results indicate that there is more than one factor with eigenvalues greater than 1, and the maximum variance explained is 39.63%. Thus, it is evident that a single factor does not account for the majority of the variance, suggesting that the issue of common method bias is not severe in this research.

### 4.3 Descriptive statistics

Table 1 displays the means, standard deviations, correlations, and reliabilities of core study variables. Customer mistreatment was found to be positively related to turnover intention (*r* = 0.40, *p* < 0.01). Hypothesis 1 is thus supported. Customer mistreatment was also found to be positively related to stress symptoms (*r* = 0.41, *p* < 0.01) and negative related to affective commitment (*r* = −0.43, *p* < 0.05). Stress symptoms was negatively associated with affective commitment (*r* = −0.41, *p* < 0.01) and positively related to turnover intentions (*r* = 0.31, *p* < 0.01). Affective commitment was negatively associated with turnover intentions (*r* = −0.53, *p* < 0.01).

### 4.4 Hypotheses testing

**Figure 2:**
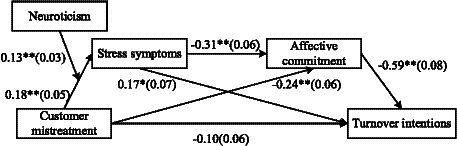
Results of structural equation modelling, Unstandardized coefficients are shown with standard errors in parentheses. For clarity, control variable paths are not pictured. ^*^p < 0.05. ^**^p < 0.01.

Figure 2 depicts SEM analysis results for the overall research model. The overall research model revealed good fit indices (CFI = 0.95, TLI = 0.94, IFI =0.95, SRMR = 0.05, RMSEA = 0.05).

To test the mediating effect of stress symptoms in the relationship between customer mistreatment and toll collectors’ turnover intentions, PROCESS model 4 in SPSS 23.0 was adopted with 5,000 bootstrap samples. Results show that the indirect effect of customer mistreatment on turnover intentions through stress symptoms is significant (*β* = 0.11, *SE* = 0.04) with a 95% confidence interval of [0.05, 0.20]. Hypothesis 2 is thus supported.

We used the same method to test the mediating effect of affective commitment in the relationship between customer mistreatment and toll collectors’ turnover intentions. Results show that the indirect effect of customer mistreatment on turnover intention through affective commitment is significant (*β* = 0.24, *SE* = 0.05) with a 95% confidence interval of [0.14, 0.35]. Hence, Hypothesis 3 is supported.

Hypothesis 4 predicts that there is a sequential mediation from customer mistreatment to toll collectors’ turnover intentions through stress symptoms and affective commitment. To test Hypothesis 4, PROCESS model 6 in SPSS 23.0 was adopted with 5,000 bootstrap samples. Results show that the indirect effect of customer mistreatment on toll collectors’ turnover intentions through stress symptoms and affective commitment is significant (*β* = 0.06, *SE* = 0.02) with a 95% confidence interval of [0.02, 0.12], which does not contain 0, indicating that Hypothesis 4 is supported.

**Figure 3:**
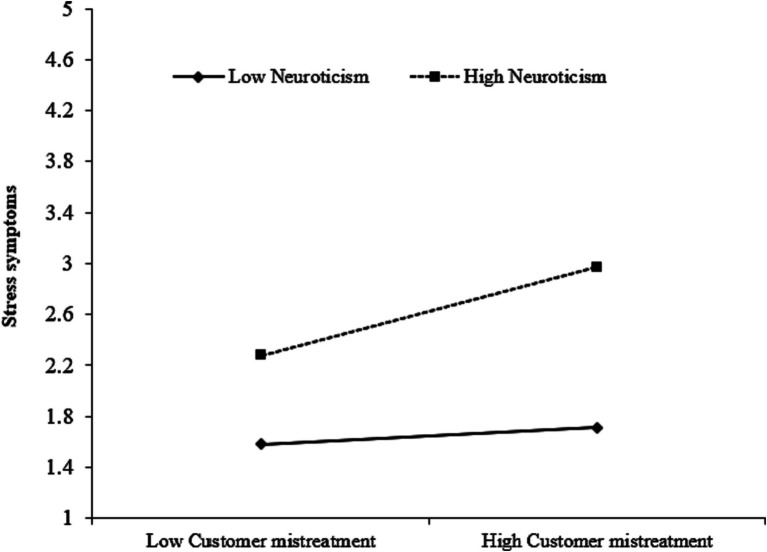
The moderating effect of neuroticism on the relationship between customer mistreatment and stress symptoms.

To test Hypothesis 5a, we constructed the latent interaction term of customer mistreatment and neuroticism. As Figure 2 shows, the interactive effect of customer mistreatment and neuroticism on stress symptoms is significant [*β* = 0.15, *SE* = 0.03, *95% CI* (0.08, 0.21), *p* < 0.01]. To aid the interpretation, we plotted the two-way interaction effect according to Preacher et al.’s (2006) method. As shown in Figure 3, the positive relationship between customer mistreatment and stress symptoms is stronger for employees in higher neuroticism. The simple slope for employees in high neuroticism (i.e., one standard deviation above the mean) is significant (*β* = 0.35, *t* = 6.32, *p* < 0.01), whereas the simple slope for employees in lower neuroticism (i.e., one standard deviation below the mean) is not significant (*β* = 0.07, *t* = 1.20, *p* > 0.05), Hypothesis 5a is thus supported.

We used the PROCESS model 83 in SPSS 23.0 to test the moderating effect of neuroticism in the indirect relationship between customer mistreatment and toll collectors’ turnover intentions through stress symptoms and affective commitment. Results showed that the indirect effect of customer mistreatment on toll collectors’ turnover intentions was significantly moderated by neuroticism [*β* = 0.02, *SE* = 0.01, *95% CI* (0.01, 0.05)]. We further examined the conditional indirect effects of customer mistreatment on toll collectors’ turnover intentions through stress symptoms and affective commitment at two values of neuroticism (i.e., one standard deviation above the mean and one standard deviation below the mean). Results shown in Table 3 indicate that when neuroticism is higher, the indirect effect is significant [*β* = 0.05, *SE* = 0.02, *95% CI* (0.02, 0.11)]; while when neuroticism is lower, the indirect effect is not significant [β = 0.01, *SE* = 0.01, *95% CI* (−0.01, 0.04)]. Thus, Hypothesis 5b is supported.

**Table 3:** Moderated mediation results for customer mistreatment across levels for neuroticism.

Moderator	Level	Turnover intentions	
		Conditional indirect effect	Bias-corrected 95% CI
Neuroticism	Low (−1 SD)	0.01	[−0.01, 0.04]
	Medium	0.03	[0.01, 0.07]
	High (+1 SD)	0.05	[0.02, 0.11]
Index of moderated mediation		0.02	[0.02, 0.11]

*N* = 230. CI, Confident intervals.

## 5 Discussion

This research contributes to the broader understanding of how toll collectors are impacted by customer mistreatment and its subsequent influence on their intention to leave. Utilizing the framework of affective events theory (Weiss and Cropanzano, 1996), this study constructs and tests a moderated mediation model to unravel why and when customer mistreatment influences toll collectors’ turnover intention. Upon analyzing responses from 230 toll collectors, the findings align with our initial hypotheses, indicating that customer mistreatment indeed acts as a catalyst, prompting toll collectors to consider leaving their jobs. This effect is mediated by an increase in stress symptoms and a decrease in affective commitment. Additionally, this study unveils that neuroticism plays a crucial role in this dynamic, acting as a moderating variable that exacerbates the adverse impacts of customer mistreatment. Specifically, toll collectors with higher levels of neuroticism experience a more pronounced negative effect of customer mistreatment on their turnover intention. These insights not only contribute to the academic discourse surrounding toll collectors’ turnover but also bear significant implications for managerial practices and strategies aimed at mitigating the adverse effects of customer mistreatment on toll collectors.

### 5.1 Theoretical implications

The current research extends the existing literature by incorporating affective event theory to broaden the investigation into the relationship between customer mistreatment and turnover intentions among service employees. In accordance with the meta-analysis conducted by Wu et al. (2023), AET provides a theoretical foundation to elucidate the mediating mechanism involving service employees’ affective outcomes in the associations between customer mistreatment and employees’ attitudinal and behavioral outcomes. AET is specifically introduced in this study to explain the association between customer mistreatment and toll collectors’ turnover intentions. This research found that stress symptoms and affective commitment can be used to explain the mechanism of customer mistreatment and toll collectors’ turnover intentions. Research shows that when toll collectors suffer from customer mistreatment, they may suffer from worse stress symptoms, lower affective commitment, and eventually highway company will face toll collectors’ turnover. This means that employees feel more terrible affective states and raise reduce emotional attachment to organization.

On the other hand, this study introduces neuroticism as a critical boundary condition that influences the relationship between customer mistreatment and turnover intention, specifically within the context of toll collectors. The identification of neuroticism underscores the importance of considering individual differences in personality when addressing issues of employee turnover (Priyadarshi and Premchandran, 2022). This is particularly relevant for toll collectors, as their day-to-day interactions with customers can vary significantly, necessitating a personalized approach to mitigate the adverse effects of customer mistreatment (Liu et al., 2022).

Finally, while research on customer mistreatment around tourism and hospitality industry is relatively common (Pu et al., 2022), special service groups are often overlooked. By zeroing in on toll collectors, this study addresses a gap in existing literature, which has often overlooked this occupational group, hence enriching our understanding of employee behavior in this specific domain (Alpu, 2015; Puswiartika et al., 2019). Toll collecting is a unique occupation, often characterized by repetitive tasks and high customer interaction, which can lead to a heightened vulnerability to mistreatment. Besides, the research further validates the affective events theory framework within diverse occupational settings, showcasing the theory’s versatility and applicability (Baranik et al., 2017). This approach encourages further occupational-specific research, promoting a trend of precision and customization in organizational psychology studies. The insights gained from focusing on toll collectors also contribute to the broader discourse on occupational health and employee well-being, as the study sheds light on the tangible ways in which customer interactions can impact an employee’s mental health and commitment to their job (Chi et al., 2018).

### 5.2 Practical implications

The current study provides valuable recommendations for management practices. First, recognizing the link between customer mistreatment and toll collectors’ turnover intentions is crucial for maintaining a healthy work environment and reducing employee turnover. We advocate training in communication and stress management for toll collectors. These programs could focus on equipping toll collectors with effective communication and conflict resolution skills, as well as strategies to manage and mitigate stress. By doing so, toll collectors may be better prepared to handle difficult interactions with customers, potentially reducing the incidence of stress symptoms and increasing their commitment to the organization, which in turn could lower their turnover intentions. Moreover, this proactive approach not only supports the well-being of the employees but also contributes to a more efficient toll collection system and a better customer experience, ultimately benefiting both the workforce and the organization as a whole.

Second, since stress symptoms mediate the relationship between customer mistreatment and turnover intentions, organizations could implement stress relief and wellness programs. Providing access to counseling services, relaxation spaces, and stress management workshops could help alleviate stress symptoms, potentially reducing turnover intentions. Developing strategies to enhance affective commitment among toll collectors could serve as a buffer against the negative impacts of customer mistreatment. Engaging in practices that increase job engagement, such as recognizing and rewarding good performance, providing opportunities for career development, and creating a supportive work environment, could strengthen employees’ emotional attachment to the organization and reduce their intention to leave. Moreover, understanding the sequential mediation from customer mistreatment to turnover intentions through stress symptoms and affective commitment can guide interventions at multiple levels. Addressing both the immediate stress responses to customer mistreatment and the longer-term implications for employee engagement and commitment could provide a more comprehensive strategy for reducing turnover intentions.

Third, recognizing that individuals have different personality traits which can influence their reactions to customer mistreatment, organizations could implement personalized intervention strategies. For toll collectors with high levels of neuroticism, who are likely to be more adversely affected by customer mistreatment, organizations could provide additional resources and support, such as access to counseling services or stress management workshops. Tailoring interventions to individual needs ensure that support is provided where it is most needed, potentially mitigating the negative impacts of customer mistreatment on stress symptoms, affective commitment, and turnover intentions.

By integrating these practical implications into their human resource management practices, organizations can create a more supportive work environment, reduce the negative impacts of customer mistreatment, and ultimately enhance the well-being and retention of their toll collectors.

### 5.3 Limitations and suggestions for future research

While this study has made valuable contributions, it is essential to acknowledge its limitations and explore potential directions for future research. First, the research sample in this study was specifically focused on examining the effects of customer mistreatment on the health (i.e., stress symptoms and affective commitment) and turnover intentions of highway toll collectors, limiting the generalizability of the findings to other job contexts or industries. Future research should aim to broaden the scope of the study by including a more diverse sample of job types and industries. This will help establish the generalizability of the effects of unfair customer treatment on employee health and turnover across various work settings.

Second, all data, including customer mistreatment, stress symptoms, affective commitment, turnover intentions, and neuroticism were collected from highway toll collectors at the same time. To mitigate this risk, we adhered to the data collection guidelines proposed by Podsakoff et al. (2003). These guidelines included ensuring confidentiality and emphasizing that there were no definitive right or wrong answers to the survey questions. While the common method bias test suggests that the influence of common method bias is not significant in this study, it is advisable for future research to proactively mitigate these concerns by employing time-lagged, longitudinal, or experimental research designs, allowing for the observation of changes over time and the identification of potential causal relationships between variables. This would provide a more accurate representation of how customer mistreatment impacts employees’ well-being and turnover intentions.

Third, based on AET, we investigated stress symptoms and affective commitment as the mechanism between customer mistreatment and turnover intention for highway toll collectors. However, other potential mechanisms may exist. For example, considering the job context, customer mistreatment may make highway toll collectors feel disconnected from their colleagues and organizations, their job embeddedness could decrease (Salem et al., 2023). Employees with high job embeddedness typically have a strong emotional attachment to their job and organization (Mitchell et al., 2001). Mistreatment can erode this attachment, causing them to consider leaving the job and, consequently, their attachment to the organization. Thus, it is essential to consider these potential mechanisms in future research to gain a more comprehensive understanding of the dynamics at play in the relationship between customer mistreatment and turnover intentions among highway toll collectors.

Fourth, we only examined the moderating role of highway toll collectors’ neuroticism, capturing the negative impact of highway toll collectors’ personality on the relationship between customer mistreatment and highway toll collectors’ turnover intention. The research model in this study did not take into account specific job characteristics relevant to highway toll collectors, such as job independence (Tsen et al., 2021), which could potentially influence the impact of customer mistreatment on highway toll collectors. Future studies should consider including job-related variables like job independence to offer a more comprehensive view of the dynamics at play in the workplace.

By addressing these limitations and following these suggestions, future research can build upon the foundation laid by this study and provide a more nuanced understanding of the impacts of customer mistreatment on employee well-being and turnover intentions.

## 6 Conclusion

Conceptualized within AET, this study builds a moderated mediation model that explains why and when customer mistreatment promotes highway toll collectors’ turnover intentions. 230 sample data provide the initial evidence that customer mistreatment is positively related to highway toll collectors’ turnover intentions through increased stress symptoms and decreased affective commitment. Moreover, highway toll collectors’ neuroticism acts as a moderator, strengthening the indirect negative impact of customer mistreatment on highway toll collectors’ turnover intentions. Our findings contribute to the literature on the relationship between customer mistreatment and turnover intentions by focusing on perspective of affective events theory and centering on highway toll collectors as research objects. We hope that this study offers insights for future research to pay more attention to the adverse impact of customer mistreatment on service employees’ turnover intentions and focus on overlooked service provider groups, such as highway toll collectors.

## Data availability statement

The raw data supporting the conclusions of this article will be made available by the authors, without undue reservation.

## Author contributions

YG: Conceptualization, Data curation, Formal analysis, Funding acquisition, Investigation, Methodology, Project administration, Resources, Software, Supervision, Validation, Visualization, Writing – original draft. YL: Conceptualization, Writing – review & editing, Data curation, Formal analysis, Methodology, Software, Supervision, Writing – original draft. JH: Conceptualization, Writing – review & editing, Investigation. JZ: Conceptualization, Investigation, Writing – original draft.

## Ethics statement

Ethical review and approval was not required for the study on human participants in accordance with the local legislation and institutional requirements. Written informed consent was not required to participate in this study in accordance with the local legislation and institutional requirements.
